# Automated high throughput animal CO1 metabarcode classification

**DOI:** 10.1038/s41598-018-22505-4

**Published:** 2018-03-09

**Authors:** Teresita M. Porter, Mehrdad Hajibabaei

**Affiliations:** 1 The Centre for Biodiversity Genomics & Department of Integrative Biology, University of Guelph, 50 Stone Road East, Guelph, ON N1G 2W1 Canada; 2 Great Lakes Forestry Centre, Natural Resources Canada, 1219 Queen Street East, Sault Ste. Marie, ON P6A 2E5 Canada

## Abstract

We introduce a method for assigning names to CO1 metabarcode sequences with confidence scores in a rapid, high-throughput manner. We compiled nearly 1 million CO1 barcode sequences appropriate for classifying arthropods and chordates. Compared to our previous Insecta classifier, the current classifier has more than three times the taxonomic coverage, including outgroups, and is based on almost five times as many reference sequences. Unlike other popular rDNA metabarcoding markers, we show that classification performance is similar across the length of the CO1 barcoding region. We show that the RDP classifier can make taxonomic assignments about 19 times faster than the popular top BLAST hit method and reduce the false positive rate from nearly 100% to 34%. This is especially important in large-scale biodiversity and biomonitoring studies where datasets can become very large and the taxonomic assignment problem is not trivial. We also show that reference databases are becoming more representative of current species diversity but that gaps still exist. We suggest that it would benefit the field as a whole if all investigators involved in metabarocoding studies, through collaborations with taxonomic experts, also planned to barcode representatives of their local biota as a part of their projects.

## Introduction

Ecological investigations, such as environmental biomonitoring, require the identification of individual specimens. This is normally done for each individual specimen by comparing suites of morphological characters with those described in taxonomic keys. In comprehensive studies that span different taxonomic groups, such as vegetation, microbes, and animals, it can be difficult to access the broad array of expertise and keys needed for identification. In large-scale studies it can also be costly and time consuming to process large numbers of samples in a timely manner. There are also difficulties in identifying damaged, partial, or immature specimens that lack the appropriate morphological characters for identification. Impediments to the study of taxonomy and the users of taxonomy have spurred the development and application of DNA-based techniques, such as marker gene surveys, coupled with high throughput sequencing for biodiversity research^1–3^.

The advantage of a marker gene approach is that it does not require the isolation or identification of individual specimens. Instead, the method can be used to survey the community of organisms present in the environmental DNA (eDNA) extracted from soil, water, or passively collected biomass. A broad array of organisms can be targeted by choosing the appropriate marker such as 16S ribosomal DNA (rDNA) for prokaryotes, ITS or LSU rDNA for fungi, or rbcL and matK chloroplast DNA for plants. When the cytochrome c oxidase (CO1) gene became the target of marker gene surveys, the approach became known as metabarcoding^3^. This approach has already been applied to agricultural, forestry, fishery, conservation, biodiversity, and biomonitoring programs^4,5^. Applied metabarcoding has become so popular because of the widespread availability of kits to extract eDNA from different substrates and the availability of high throughput sequencing.

With DNA metabarcoding, taxonomic assignment in new studies is shifted to computational algorithms. Indeed, the method is entirely reliant on having DNA barcode sequences from curated samples that have already been identified. So the method does not entirely relieve the field from the taxonomic impediment, but it does improve accessibility to existing named DNA barcode sequences that ideally come from curated specimens. Though there are many options available for making taxonomic assignments, most CO1 metabarcodes are still routinely assigned using the familiar top BLAST hit method^6^. This is not an ideal situation since the top BLAST hit method has already been shown to be misleading^7^, has a high false positive rate of assignment^8^, is slow, and provides no statistical measure of confidence for taxonomic assignments^9^.

The Ribosomal Database Project (RDP) classifier uses a naïve Bayesian approach to make taxonomic assignments^10^. The RDP classifier was originally developed using prokaryote 16 S rDNA sequences. The classifier can be trained, however, to make taxonomic assignments using any DNA marker. For example, the tool can also be used to classify fungi based on their ITS or LSU rDNA sequences^11^. One advantage of using the RDP classifier over the more widely used top BLAST hit method is speed. This method is much faster and can process large datasets from high throughput sequencing in a fraction of the time that it would take with BLAST^9^. This is especially important in large-scale biodiversity and biomonitoring studies where data sets can become very large. Additionally, unlike BLAST, the RDP classifier was specifically developed to provide a measure of confidence for assignments at each rank in the taxonomic hierarchy. This is a key aspect of the method as it allows users to easily summarize results to more inclusive ranks, where necessary, as well as filter for good results based on bootstrap support values. The RDP classifier is open-source, well-documented, and has a long history of use in related fields^12^.

Although a variety of resources exist for taxonomically assigning prokaryotes and fungi^11–17^, most CO1 metabarcodes are still assigned using BLAST. The BOLD CO1 database was designed as a curation and analysis tool for individual specimens and it is not suitable for the analysis of the large batches of CO1 metabarcodes generated by high throughput sequencing^18^. To address that gap, we previously developed an Insecta CO1 training set that could be used with the RDP classifier. Unfortunately, it could not be used to identify non-insect animals^9^.

The purpose of this study is to introduce the CO1 metabarcoding community to an alternative method for taxonomically assigning CO1 metabarcodes. (1) We compiled a comprehensive training set for the RDP classifier focusing on the Chordata and Arthropoda, the two largest groups of publicly available CO1 sequences. (2) We benchmark the performance of the classifier for a range of sequence lengths and taxa with a focus on groups important for freshwater biomonitoring. (3) We provide guidelines for bootstrap support cutoffs. (4) We show that the RDP classifier is faster than the top BLAST hit approach and has a lower false positive rate (FPR). (5) We also show the improvement in insect CO1 classification and reference set coverage from 2013 to 2016. Altogether we show that this approach is a significant improvement over the widely used top BLAST hit method for CO1 metabarcode taxonomic assignments.

## Results

In this study we introduce a new CO1 training set suitable for classifying the broad range of arthropods and chordates commonly found in metabarcode studies geared towards ecological assessments and biomonitoring. Improvements include more than three times the taxonomic coverage, outgroup taxa to flag other major eukaryote taxa, and a more comprehensive training set based on almost five times the number of sequences. The term ‘training set’ refers to the set of files produced after we trained the classifier to make CO1 taxonomic assignments. Training sets can be used directly with the RDP classifier and no further training is needed from the user. Our main results are the v1 and v2 training sets that can be used to make genus- and species-rank taxonomic assignments, respectively. The taxonomic composition of the CO1 Eukaryote v1 training set is summarized in Table 1 and in detail in Table S1. A similar summary table is shown for the CO1 Eukaryote v2 training set in Table S2. Outgroup taxa were also included to help sort non-Arthropod and non-Chordata taxa into broad groups such as fungi, diatoms, or nematodes.

**Table 1:** CO1 Eukaryote v1 training set summary.

Training set	Number of taxa (all ranks)	Number of sequences
Whole training set	29,998	912,253
Arthropoda	21,267	685,651
Chordata	7,344	215,530
Outgroup taxa	1,385	11,072

**Figure 1:**
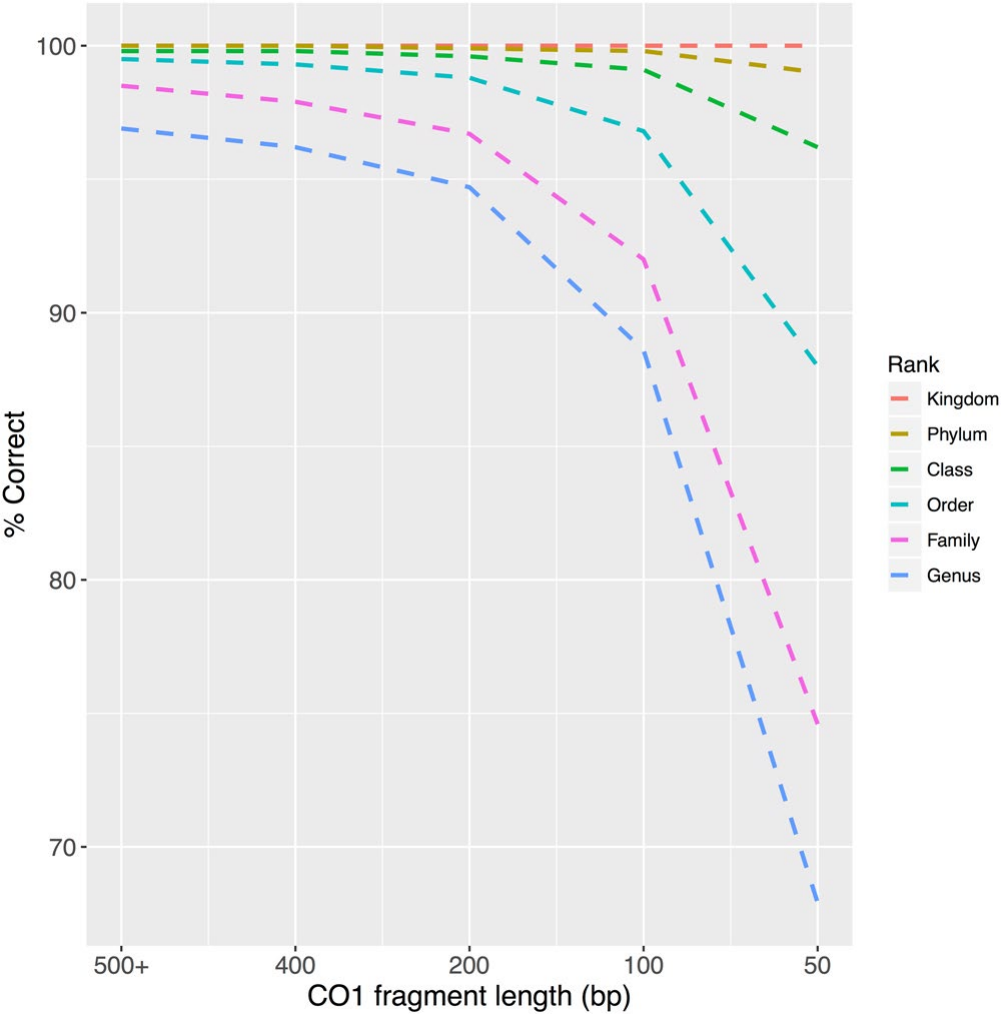
The proportion of correct taxonomic assignments increases with more inclusive taxonomic ranks and longer CO1 sequences. Results summarize results from leave-one-out testing of the CO1 Eukaryote v1 training set.

The proportion of singletons in the dataset can indicate the presence of groups with low coverage and so is summarized for both the v1 and v2 training sets in Table S3. The proportion of singleton genera in the genus-trained classifier is 23% compared with the proportion of singleton species in the species-trained classifier at 33%. In this study we define a false positive (FP) as an incorrect taxonomic assignment with a bootstrap support value greater than the cutoff. To avoid making FP assignments, the bootstrap cutoffs presented in this study should be treated as *minimum* cutoff values. Taxa for the training set were sampled to emphasize Arthropoda and Chordata since these were the best-represented eukaryote phyla in the GenBank nucleotide database. Figure 1 shows the proportion of correctly assigned sequences for a variety of query lengths at a variety of taxonomic ranks. Since the classifier is not meant to classify taxa not represented in the database, leave-one-out testing results from singletons were excluded from this figure and no bootstrap support cutoff was used. Classifier accuracy is highest at more inclusive taxonomic ranks, especially for fragments 200 bp or longer.

The receiver operator characteristic (ROC) curves for full length CO1 barcode sequences identified to various taxonomic ranks and a range of fragment lengths at the genus rank is shown in Figure S1. A ROC curve shows the relationship between the false positive rate (FPR) and the true positive rate (TPR) as the bootstrap support cutoff is tuned from 0 to 100%. The FPR represents the proportion of incorrect assignments with a high bootstrap support value out of all incorrect assignments. The TPR represents the proportion of correct assignments with a high bootstrap support value out of all correct assignments. In these figures, the bootstrap cutoff values are not directly shown on the plot, rather we show the resulting change in TPR and FPR. Generally, lower cutoffs result in lower TPRs (and FPRs) and higher cutoffs result in higher TPRs (and FPRs). The optimal place to be when choosing a cutoff value is in the top left quadrant where the TPR is high and the FPR is low. In this figure, the ROC curves are calculated from leave-one-out testing results where singletons were excluded. Points lying above the 50% line indicate results better than those obtained by chance. The high area-under-the-curve values indicate high true positive rates and good classifier performance across a wide range of bootstrap support values. In the top figure, the TPR for full length CO1 sequences at all taxonomic ranks is high indicating that most of the assignments are correctly assigned at any cutoff value. In the bottom figure, TPRs increases with longer CO1 fragment lengths.

Since classification performance varies with fragment size and taxonomic assignment rank, we have calculated a matrix of minimum bootstrap support value cutoffs to obtain 99% correct assignments during leave one out testing (Table 2). The assumption here, as well as when using the top BLAST hit method, is that the query sequence is actually represented in the database. Singletons were excluded from this analysis so cutoffs are based on 77% of the sequences in the original CO1 Eukaryote v1 training set. Also shown is the corresponding reduction in the proportion of classified sequences after applying the minimum bootstrap support cutoff values. A similar table for the Eukaryote v2 classifier trained to the species rank is shown in Table S4. Generally as the amount of sequence information decreases with decreasing CO1 sequence length, higher bootstrap support cutoff values are needed to observe 99% correct assignments. Similarly, as assignments are made to increasingly specific ranks, higher cutoff values are required to observe 99% correct assignments.

**Table 2:** Bootstrap support cutoff values that produced at least 99% correct assignments during CO1 Eukaryote v1 leave-one-out testing.

Rank	500 bp+	400 bp	200 bp	100 bp	50 bp
	**Minimum bootstrap support cutoff (%)**				
Superkingdom	0	0	0	0	0
Kingdom	0	0	0	0	0
Phylum	0	0	0	0	0
Class	0	0	0	0	60
Order	0	0	10	40	80
Family	20	20	30	40	80
Genus	70	60	60	60	N/A
	**Reduction of sequences classified after applying minimum bootstrap support cutoff (%)**				
Superkingdom	0.0	0.0	0.0	0.0	0.0
Kingdom	0.0	0.0	0.0	0.0	0.0
Phylum	0.0	0.0	0.0	0.0	0.0
Class	0.0	0.0	0.0	0.0	13.2
Order	0.0	0.0	0.2	5.7	60.7
Family	0.7	1.2	4.1	17.1	78.7
Genus	3.4	4.7	10.8	31.0	N/A

‘N/A’, not applicable, refers to the inability to observe 99% correct taxonomic assignments.

Applying a bootstrap support cutoff can reduce the proportion of incorrect taxonomic assignments. Figure S2 shows the proportion of incorrect assignments for Arthropoda sequences both with and without using bootstrap support cutoffs. Leave one out testing results from singletons were included here to simulate the taxonomic assignment of sequences without congenerics in the database. The 70% bootstrap support cutoff value was selected for full length (500 bp+) CO1 sequences as shown in Table 2. When classifying Arthropoda sequences, 23% of which were known to have no congenerics in the training set (Table S3), applying a 70% bootstrap support cutoff at the genus rank reduced the misclassification rate for nearly all classes to ~1% while reducing the number of assigned sequences by ~3%. A similar analysis with Chordata is shown in Figure S3. The proportion of incorrect assignments for all phyla, Arthropoda and Chordata classes, as well as for the orders in the large Insecta and Actinopteri groups are shown in the Tables S5–S9. When we focus on groups that are particularly important in freshwater biomonitoring, we see that database sequences are highly skewed towards Diptera and that although the proportion of incorrect classification varies across groups the application of a bootstrap support cutoff reduces these rates to ~1% incorrect assignments (Table 3). One exception is for sequences in Megaloptera that have a higher proportion of incorrect assignments (1.7%) even after using a 70% bootstrap support cutoff at the genus rank for full length (500 bp+) CO1 sequences. These tables show how database representation and misclassification rates can vary across taxonomic groups.

**Table 3:** Representation of freshwater biomonitoring taxa in the Eukaryote CO1v1 training set.

Class	Order	No. reference sequences	% Incorrect (No cutoff)	% Incorrect (Cutoff)
Bivalvia	—	667	3.7	0.3
Clitellata	—	N/A	N/A	N/A
Gastropoda	—	1,896	3.7	0.4
Insecta	Coleoptera	89,484	7.5	1.1
Insecta	Diptera	118,896	3.8	0.8
Insecta	Ephemeroptera	6,722	2.8	0.3
Insecta	Megaloptera	469	3.6	1.7
Insecta	Odonata	3,553	6.9	1.2
Insecta	Plecoptera	2,679	2.7	0.1
Insecta	Trichoptera	17,277	3.1	0.3
Malacostraca	Amphipoda	8,483	3.4	1.3
Malacostraca	Isopoda	3,659	2.9	0.1
Polychaeta	—	888	2.8	0.2
Turbellaria	—	N/A	N/A	N/A

N/A, not applicable, as of October 2016 there are no full length CO1 sequences identified to the species rank in the GenBank nucleotide database. We used a 70% bo otstrap support cutoff value at the genus rank.

**Figure 2:**
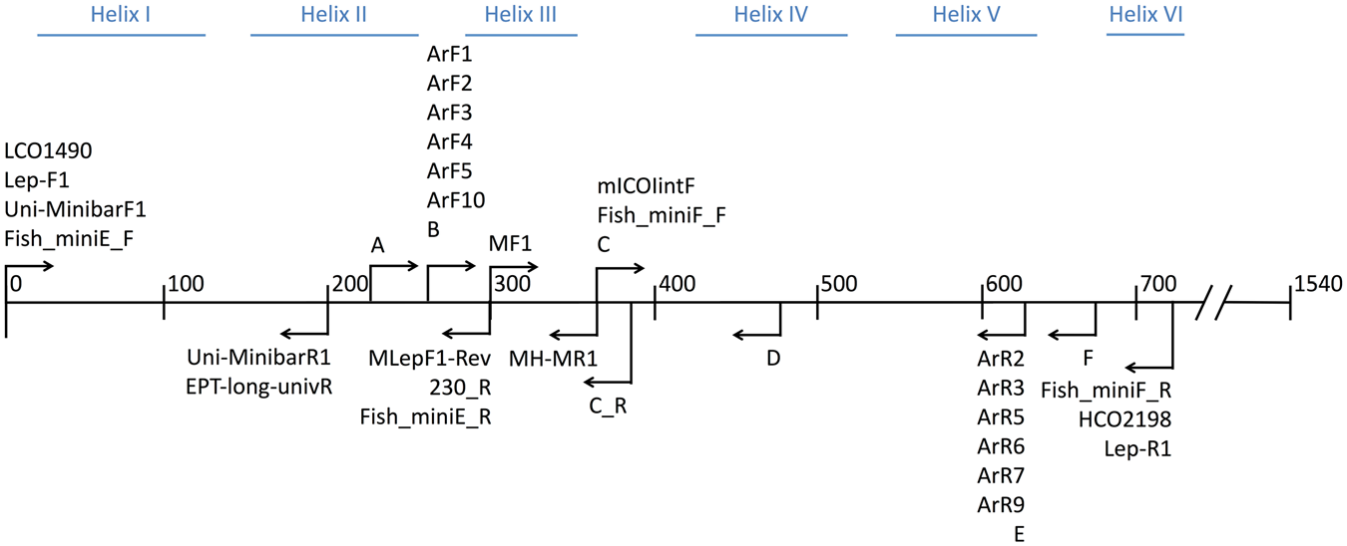
CO1 primers included in this study. Primer map of the CO1 barcoding region showing the relative position and direction of the primer-anchored 200 bp fragments analyzed in this study. The CO1 helix regions that are embedded in the mitochondrial inner membrane are also shown for reference.

**Figure 3:**
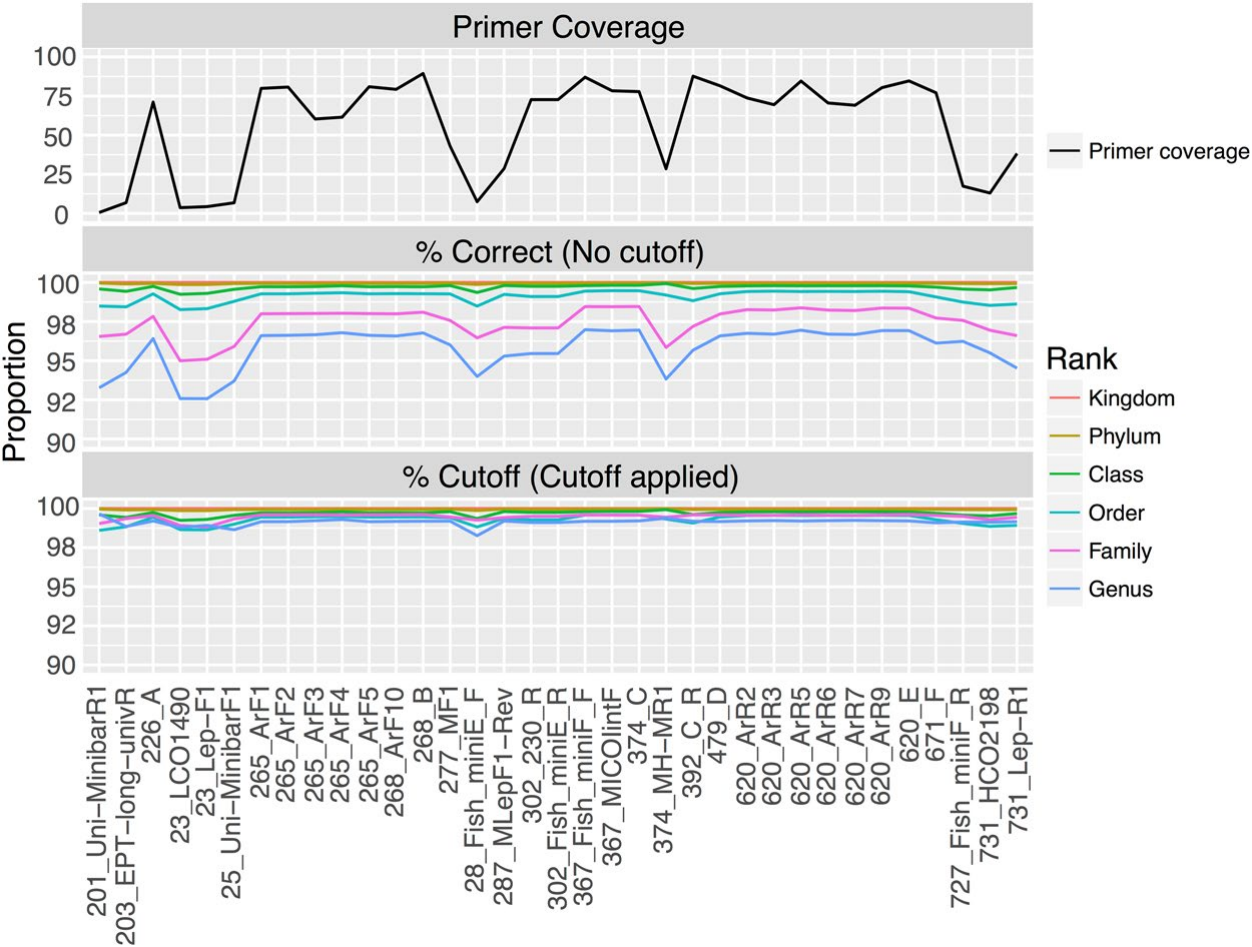
The proportion of correctly assigned primer-anchored 200 bp sequences can vary across the CO1 barcoding region before applying a bootstrap support cutoff. Primer names are prefixed with the outermost alignment position along the CO1 barcoding region and are arranged along the x-axis in the order that they would be encountered from the 5′ to 3′ end. Top panel: Coverage of primer-anchored 200 bp sequences in the CO1 Eukaryote v1 training set. Middle panel: Proportion of correct taxonomic assignments. Bottom panel: Proportion of correct assignments after filtering by a 60% bootstrap support cutoff at the genus rank. Note the differing scale on the y-axes.

Classification performance may also vary for partial CO1 sequences whether they are sampled randomly from across the barcoding region (as in Fig. 1) or if they are anchored by CO1 primers (Fig. 2). The coverage of primer-anchored 200 bp sequences sampled from the dataset varies across the length of the barcoding region. Since primers are often trimmed before submission to GenBank, it was not surprising that the Folmer barcoding primers, and other primers designed near the 5′ and 3′ end of the barcoding region, had especially low coverage in our training set (Fig. 3). The proportion of correct assignments of primer-anchored 200 bp sequences with and without 60% bootstrap support (Table 2) is also shown. Singletons were not included in this analysis. The proportion of correct assignments is especially high at the order to kingdom ranks. After applying the bootstrap support cutoff, the proportion of correct taxonomic assignments rose to ~99% across all primers at the genus, family, and order ranks. Figure 3 shows that there are minimal differences in CO1 classification performance across the barcoding region that comprises 6 alpha helices and 5 loop regions. This is in contrast with other popular metabarcode markers such as prokaryote 16 S and fungal ITS rDNA markers that show distinctive stem-loop secondary structure with peaks and troughs of classification accuracy across variable domains (loops) and conserved regions (stems), respectively^10^.

A comparison of taxonomic assignment outcomes using the top BLAST hit method and the RDP classifier with the CO1 Eukaryote v1 training set is shown for all primer-anchored 200 bp fragments in Table 4 and Figure S4. Using BLAST, no hits were returned for some queries because the expect value (e-value) was greater than the default cutoff of 10. In contrast, using the RDP classifier, a result was returned for every query. Assignment accuracy (Table 4) is highest for the top BLAST hit method, however, the FPR is ~3 times higher for BLAST than for the RDP classifier. This is significant because in this example, 397,820 taxonomic assignments are classified as ‘good’ based on the top BLAST hit metrics but they are actually incorrect. In general, using the RDP classifier with the CO1 Eukaryote v1 training set and the recommended minimum bootstrap support cutoff at the genus rank significantly reduces the FPR.

**Table 4:** Taxonomic assignment outcomes at the genus rank from primer-anchored 200 bp sequences using the top BLAST hit method compared with the RDP classifier and the CO1 Eukaryote v1 training set.

Method	N*	No results returned**	TP	FN	TN	FP	Accuracy	TPR	FPR
Top BLAST hit approach	17,960,965	1,642	17,559,411	3,350	384	397,820	98%	~100%	~100%
RDP Classifier CO1 Eukaryote v1	17,962,607	N/A	16,887,619	727,269	230,262	117,457	95%	96%	34%

TP = true positive, FN = false negative, TN = true negative, FP = false positive, TPR = true positive rate, FPR = false positive rate

~Indicates that the value was rounded up and is nearly 100%

*N = Total number of primer-anchored 200 bp CO1 sequences used as queries

**BLAST results were not returned because the expect value was greater than 10.

**Figure 4:**
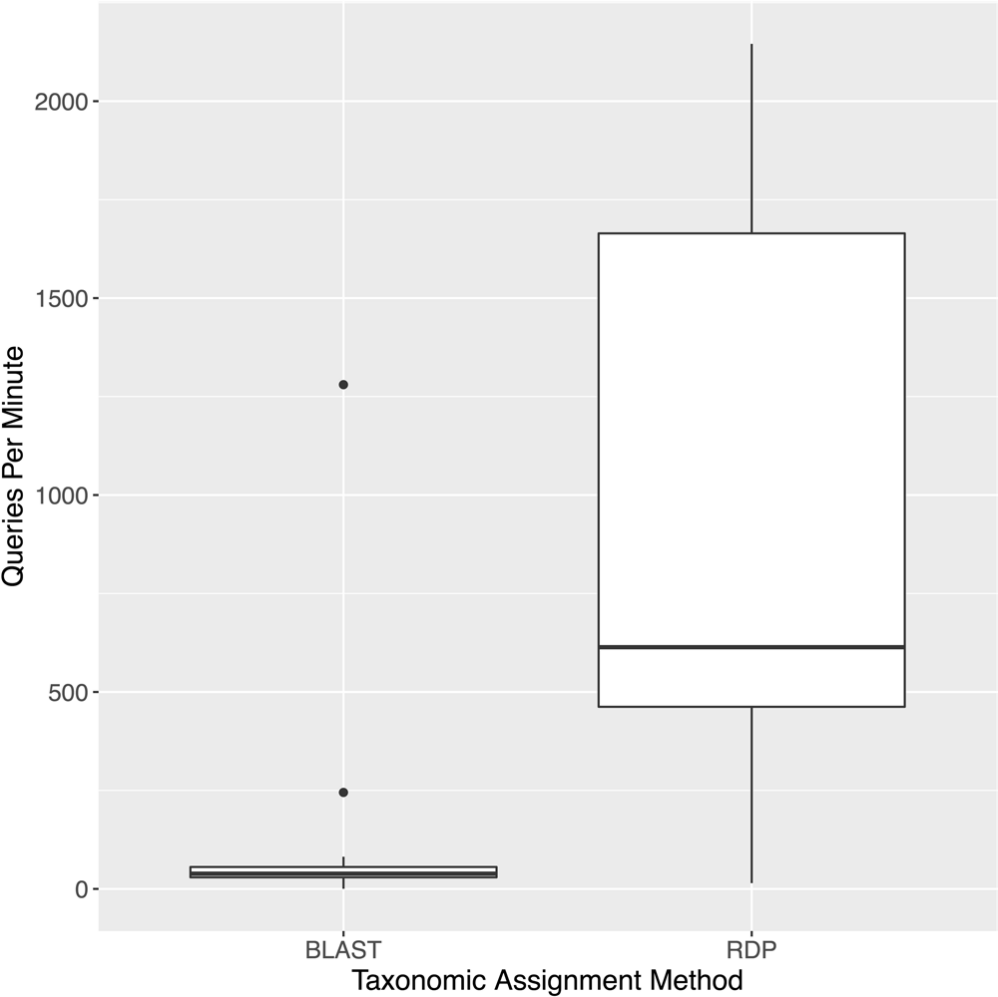
The RDP classifier taxonomically assigns more queries per minute than the top BLAST hit method. The number of primer-anchored 200 bp query sequences taxonomically assigned per minute is compared using the top BLAST hit method against a locally installed copy of the nucleotide database and the RDP classifier 2.12 with the CO1 Eukaryote v1 training set.

We also compared the time needed to make high-throughput sequence-based taxonomic assignments using the top BLAST hit method and the RDP classifier (Fig. 4). Using a single processor, making assignments using the RDP classifier with the CO1 Eukaryote v1 training set was on average ~19 times faster than using the top BLAST hit method. We did not consider the extra time needed to process tabular BLAST output into a usable format by adding taxonomic lineages and calculating query coverage.

Compared with a 2013 training set, we found ~3 times more class Insecta reference sequences 500 bp + identified to the species rank (561,841 versus 190,333) and one additional order ‘Zoraptera’ from GenBank (Table S10). The group of top five Insecta orders with the greatest number of reference sequences has not changed from 2013 to 2016, though each group contains many more reference sequences today than 3 years ago (Table S11). The bottom five Insecta orders with the least number of reference sequences has changed slightly from 2013 and in the current training set includes Grylloblattodea (n = 1), Zoraptera (n = 2), undef_Insecta (n = 9), Mantophasmatodea (n = 30), and Dermaptera (n = 37) (Table S12). As expected, as the number of reference sequences in the database grows, the proportion of genus rank incorrect assignments decreases (Table S13). Representation of various Insecta orders is shown in detail for CO1 sequences in the large class Insecta (Table S8). To further reduce misclassification rates in class Insecta, reference sequences for the Grylloblattodea, Zoraptera, Lepidotrichidae, Lepismatidae, Nicoletiidae, Mantophasmatodea, and Dermaptera need to be added to public databases.

## Discussion

Metabarcoding is widely used to survey bacteria using 16 S rDNA, fungi using ITS rDNA, plants using rbcL + matK cpDNA, as well as animals using CO1 rDNA markers. Bioinformatics, specifically taxonomic assignment methods, are a moving-target in this rapidly developing field. There are broad communities already devoted to developing 16 S and ITS rDNA resources^12,14–16^. In the field of CO1 metabarcoding there is plenty of room for newer algorithms and method development. An advantage of CO1 metabarcoding is the ability to directly compare eDNA metabarcodes with real communities with tractable and quantifiable richness using morphology-based methods. Similar comparisons are a difficult feat for hyper-diverse microbial communities where base-line richness is often unknown. This provides an excellent foundation for benchmarking the performance of new methods.

CO1 metabarcoding has been extensively compared with morphology-based biomonitoring methods across a range of applications and has been repeatedly shown to detect more and/or a complementary suite of taxa compared with traditional methods^4^. The continued interest and growing popularity of DNA metabarcoding in a diverse array of fields is driven by the scalability of this method when coupled with high throughput DNA sequencing^19^. Detection of taxa especially important for biomonitoring relies on standardized, representative, and reproducible field sampling methods such as those developed by the Canadian Aquatic Biomonitoring Network (CABIN) or the Australian River Assessment Scheme (AUSRIVAS)^20,21^. Improvement of lab methods such as primer development for PCR, the use of multiple markers to increase detection coverage, and the development of PCR-free methods are active areas of research^6,22–24^. Despite all this work to validate CO1 metabarcoding, a commonly overlooked area has been at the taxonomic assignment step.

Too often, it has been accepted without question that a generic top BLAST hit approach is sufficient to make high throughput CO1 taxonomic assignments. Unfortunately, the BLAST metrics commonly used for delimiting good taxonomic assignments such as percent identity, query coverage, bit score, e-value, or combinations thereof simply provides different measures of similarity to a top hit and a measure of random background noise in the database^25^. The RDP classifier, on the other hand, was developed specifically to make taxonomic assignments from marker gene sequences and provide a measure of confidence to assess how likely the assignment is to be correct^10^. With the top BLAST hit method, if there is no top BLAST hit that meets the user’s criteria for a good assignment, then no assignment can be made. With the RDP classifier, if there are no congenerics in the database or if the genus rank assignment has a low confidence score, it may still be possible to make an assignment to a more inclusive rank if there are, for example, confamilial sequences in the database.

The impact of different kinds of taxonomic assignment errors has been discussed in the literature^8^. One particular concern is the effect of false positive taxonomic assignments. In this study, a false positive was defined as a sequence taxonomically assigned with high confidence even though it is wrong. This is especially significant when the cost of making a misidentification is high. A false positive assignment, for example, could lead investigators to over-estimate the presence or distribution of a rare threatened or endangered species^26^ or the assignment may a create false alarm for an invasive or harmful species. In such cases, the RDP classifier is a more reliable tool to use than BLAST.

Another contributor to the false positive rate is high confidence taxonomic assignments made to incorrectly identified entries in public databases. The question of annotation accuracy in GenBank is a known issue^27^. For example, it has been estimated that fungal ITS sequences in GenBank may be incorrectly identified to the species rank about 20% of the time^28^. It is reasonable to assume that similar issues also affect CO1 sequences in GenBank. With respect to taxonomic assignment, one way to circumvent this problem is to summarize taxonomic assignments to the genus or other more inclusive ranks. With the top BLAST hit method, similarity statistics represent a single query-reference sequence comparison. In this situation, if the top BLAST hit record has an incorrect identification then the taxonomic assignment will be a false-positive if the BLAST metrics meet the user’s cutoff values. With the RDP classifier, k-mer frequency profiles are based on all the sequences for each unique genus in a genus-trained classifier. In this situation, a few erroneous k-mers and frequencies are unlikely to have a large effect if most of the taxa are correctly identified. For a taxon that is poorly-represented in the database by just one or a few sequences, however, the presence of a few misidentified sequences would have a greater effect on the RDP classifier making a false-positive more likely. For this reason, in cases were species-level taxonomic assignments are needed, the user needs to be aware of the database coverage of their taxa of interest. Generally, the responsibility for ensuring that high quality CO1 reference sequence datasets are available falls to the user-community. For example, the prokaryote 16 S rDNA SILVA and Greengenes reference sets as well as the fungal ITS UNITE rDNA database were developed and are maintained by researchers with a stake in the field and they provide a tremendous resource for the broader user-community^14–16,29^. These researchers mine data from GenBank but also subject the data to 3^rd^ party curation to provide better quality reference sets. For the animal CO1 community, GenBank entries with the BARCODE keyword indicate high quality records^18^.

In this study a false negative was defined as a sequence correctly classified but with a confidence score below the threshold cutoff. The RDP classifier is more prone to FN’s than BLAST. Type II error also encompasses the outcome when a sequence cannot be classified because congeneric sequences are missing from the database. For example, this latter scenario is of particular concern in quarantine situations that could result in the introduction of parasites, pathogens, or invasive species^8,30^. In theory, in a quarantine situation where a limited suite of taxa is of interest, it should be easier to compile a representative database. As databases grow, high confidence assignments should improve and the rate of false negatives due to missing congenerics in the database should be reduced.

If every investigator involved in a metabarcoding study could further collaborate with taxonomic experts to identify a few representatives of their local biota, this could expedite the process of making public databases more representative. For example, previous work has shown that as few as 12% of described extant Insecta genera (8,679/72,618) are currently represented by full length (500 bp+) CO1 barcode sequences identified to the species rank in the GenBank nucleotide database^9^. Querying the database three years later, we found out that 22% of extant Insecta genera (16,285/72,618) are now represented by full length sequences identified to the species rank in the GenBank nucleotide database. At this rate of growth it could take 27 more years for all currently described extant Insecta genera to be represented by a full length CO1 barcode sequence in GenBank, not counting taxa still waiting to be described. This is only the tip of the metaphorical CO1 barcode iceberg, however, as the number of insect species is exponentially higher than genera and CO1 sequence representation in databases is expected to be even less at the species rank. This data gap could have significant implications to leverage the full potential of CO1 metabarcoding in current studies. We suggest that an immediate way to improve the number of high confidence assignments for current studies is to sequence CO1 barcodes for common representatives of local biota to supplement existing databases. Even if they are not identified to the species rank, they will still represent local biota that can be targeted for further study if they prove to be of interest.

Developments in the field of CO1 taxonomic assignment are mostly geared to the assignment of single queries though some methods can assign batches of sequences at once. Methods range from tools that use HMM alignment followed by a linear search^18^, Neighbor-joining analysis^2^, BLAST^31^, minimum distance and fuzzy set theory^32^, the coalescent^33^, segregating sites^34^, neural networks^35^, and support vector machines^36^. Other than the first three methods, none of the alternative methods have caught on for CO1 taxonomic assignment most likely because the average user is not aware they exist or there is no portal to allow for easy implementation. The RDP naïve Bayesian classifier can be used to make taxonomic assignments in large batches. Compared to our previous Insecta CO1 classifier, the current CO1 Eukaryote training sets we describe here are suitable for classifying the broad range of arthropods and chordates commonly found in metabarcode studies geared towards ecological assessments and biomonitoring. Our method leverages the well-known RDP classifier and adapts it for use to classify animal CO1 mtDNA. Aside from the capabilities demonstrated in this study, we believe the long history of this method, open-source availability, and clear documentation will help widespread application of this method for fast and accurate high throughput taxonomic assignments. Future work involves making this method available through a web portal.

## Methods

Three sets of CO1 reference sequences were assembled: 1) Arthropoda, 2) Chordata, and 3) outgroup taxa as described below using Perl with BioPerl modules and the Ebot script^37,38^. The following search terms were used to query the NCBI taxonomy database: 1) “Arthropoda”[ORGN] AND “species”[RANK] [Aug. 10, 2016], 2) “Chordata”[ORGN] AND “species”[RANK] [Aug. 24, 2016], and 3) “cellular organisms”[ORGN] AND “species”[RANK] NOT (“Arthropoda”[ORGN] OR “Chordata”[ORGN]) [Oct. 24, 2016]. A formatted taxon list was created using only taxa with complete binomial species names excluding the names containing sp., nr., aff., and cf. The NCBI nucleotide database was queried using the Entrez search term “cox1[gene] OR coxI[gene] OR CO1[gene] OR COI[gene] AND” the formatted taxon lists from above. For the outgroup taxa, the additional term “BARCODE”[keyword] was used. Sequences were retained if they were at least 500 bp and multiple sequences per species were retained when available. The associated taxonomic lineage was retrieved for each sequence. Human contaminant sequences were identified using BLAST and a custom database comprised of only human CO1 sequences. The taxonomic reports of hits with high query length coverage and high percent identity to known human sequences were individually explored, removed where necessary, and reported to NCBI. The Arthropoda, Chordata, and outgroup taxa were combined to create the CO1 Eukaryote v1 set trained to the genus rank and used with the RDP classifier v 2.12 for leave-one-out testing, cross-validation testing, and classifier training. A CO1 Eukaryote v2 training set was also created using the same sequences from above but was trained to the species rank. These training sets can be downloaded from https://github.com/terrimporter/CO1Classifier and can only be used with the command-line version of the RDP classifier that can be downloaded separately from https://sourceforge.net/projects/rdp-classifier/.

Since metabarcoding samples often contain partially degraded eDNAs, shorter fragments are often targeted to increase PCR and sequencing success. As a result, leave-one-out testing was performed for full length (500 bp+) CO1 sequences as well as for 400 bp, 200 bp, 100 bp, and 50 bp fragments. During leave-one-out testing, a sequence is removed from the dataset before it is classified. An assignment is scored as correct if the assignment matches the known taxonomy for the sequence. This assignment is made using a full set of 8 bp ‘words’ subsampled from the query sequence. Bootstrap support is assessed by randomly subsampling some of the 8 bp ‘words’ from the query sequence, making an assignment based on this set of 8 bp ‘words’, and repeating this procedure 100 times. The proportion of times the original taxonomic assignment is recovered becomes the reported bootstrap support value for the assignment. The sequence is returned to the training set and the next sequence is removed, classified, and so on. The purpose of this type of testing is to assess classifier performance.

CO1 primers from the literature, especially those targeting invertebrates or developed especially for metabarcoding eDNA were compiled. Primers tested in this study and their references are shown in Table S14. These primers were aligned against the *Drosophila yakuba* CO1 region obtained from GenBank accession X03240 using Mesquite v 3.10^39^. CO1 secondary structure features from *Bos taurus* were obtained from UniProt accession P00396. We used CUTADAPT v1.10 to retrieve primer-trimmed sequences using default settings (allowing up to a 10% mismatch in the primer sequence) from our CO1 training set in the same way that real raw sequence data would be processed with the default settings^40^. These sequences were trimmed to 200 bp fragments to simulate the average length of an Illumina read after primer trimming and we assessed assignment accuracy and coverage using leave-one-out and cross-validation testing. For each primer, the RDP classifier was directly compared with the top BLAST hit method. Assignments were compared at the genus rank for each method. ‘Good’ assignments for the RDP classifier were defined according to Table 2 for 200 bp fragments at the genus rank, requiring a bootstrap proportion of 0.60 or greater. ‘Good’ assignments for the top BLAST hit method was defined by having a top BLAST hit with percent identity >  = 95% and a top BLAST hit alignment that spans >  = 85% of the original query sequence length (query coverage). We measured the proportion occurrence and rate of different types of taxonomic assignment outcomes as defined in Figure S5.

We also compared how class Insecta sequence database composition and incorrect taxonomic assignment distribution across insect orders have changed over the past three years. This was done by comparing the proportion of incorrect assignments from class Insecta in the current CO1 Eukaryote v1 training set [August 2016] with the Insecta Genbank-Genus training set [March 2013] that both used the leave-one-out testing method provided by the RDP classifier tool^9^.

### Data availability

The taxonomy and FASTA files used for training, the final trained sets ready to be used with the RDP classifier, as well as general usage instructions are available on GitHub https://github.com/terrimporter/CO1Classifier.

## Electronic supplementary material

Supplementary Information
