# Supplementary material for: Automated high throughput animal CO1 metabarcode classification
Source: Sci Rep. 2018 Mar 9;8:4226. doi: 10.1038/s41598-018-22505-4 (PMC5844909; doi:10.1038/s41598-018-22505-4)
Supplement: Supplementary file 1 — Supplementary Information [file 41598_2018_22505_MOESM1_ESM.docx]

**Automated high throughput animal CO1 metabarcode classification**

Teresita M. Porter, Mehrdad Hajibabaei

**Supplementary Information**

**Supplementary Figures**

Figure S1: Receiver operator characteristic curves show good classifier performance across a range of taxonomic ranks for the longest CO1 sequences. Top panel: Comparison of false positive rates (FPR) and true positive rates (TPR) among full length (500 bp+) CO1 fragments classified to various taxonomic ranks. Bottom panel: Comparison among genus rank assignments for a variety of CO1 sequence lengths. A 50% chance line is shown as a grey dashed line. Area under the curve (AUC) values are shown in the legend.

Figure S2: Using bootstrap cutoffs as a filter reduces the proportion of incorrect taxonomic assignments for full length (500 bp+) CO1 Arthropoda sequences. Top panel: The proportion of incorrect assignments from leave-one-out testing is shown. Bottom panel: The proportion of incorrect assignments is shown when a bootstrap support cutoff of 70% at the genus rank is used as a filter. The grey dashed line shows the 5% incorrect assignment cover line. The size of the circles shows the total number of misclassified assignments for each Arthropoda class. Results were summarized from leave-one-out testing including singletons. The category ‘undef_Arthropoda’ includes sequences from the orders Pauropoda and Symphala as well as the genus *Prionodiaptomus*.

Figure S3: Applying a bootstrap support cutoff does not reduce the proportion of incorrect taxonomic assignments for under-sampled classes in the Chordata. Top panel: Proportion of incorrect taxonomic assignments for all full length CO1 Chordata sequences during leave-one-out testing including singletons. Bottom panel: Proportion of incorrect taxonomic assignments when a bootstrap support cutoff of 70% at the genus rank is used as a filter. The grey dashed line shows the 5% incorrect taxonomic assignment cover line. The size of the points shows the total number of misclassified assignments per Chordata class. Class Appendicularia is represented by one sequence and Thaliaceae is represented by two sequences in the Eukaryote CO1 v1 training set. The taxon ‘undef_Chordata’ contains sequences from the orders Ceratodontiformes, Coelacanthifores, Crocodylia, Lepidosireniformes, Myxiniformes, Petromyzontiformes, Sphenodontia, Squamata, Testudines, and the family Branchiostomidae.

Figure S4: Summary of taxonomic assignment outcomes from primer-anchored 200 bp CO1 sequences using the CO1 Eukaryote v1 training set. A 1:1 line is shown as a grey dashed line. Note the differing limits on the axes for each outcome. Results are from leave-one-out testing including singletons.

Figure S5: A matrix that summarizes taxonomic assignment outcomes, the terms used in this study, and the relationships between them. Abbreviations are as follows: True positive (TP), false positive (FP), false negative (FN), true negative (TN), true positive rate (TPR), false positive rate (FPR), false negative rate (FNR), true negative rate (TNR).

* The cutoff used to determine a high confidence assignment will depend on the fragment length and rank of assignment.

**Supplementary Tables**

Table S1: Taxonomic breakdown of the CO1 Eukaryote v1 training set.

| **Whole training set** | | **Arthropoda** | | **Chordata** | |
| --- | --- | --- | --- | --- | --- |
| **Phylum** | **No. sequences** | **Class** | **No. sequences** | **Class** | **No. sequences** |
| Arthropoda | 685,651 | Insecta | 561,841 | Actinopteri | 91,744 |
| Chordata | 215,530 | Arachnida | 52,328 | Mammalia | 70,397 |
| Unassigned phylum* | 3,079 | Malacostraca | 39,622 | Aves | 25,150 |
| Mollusca | 3,017 | Maxillopoda | 14,548 | Amphibia | 10,154 |
| Echinodermata | 2,504 | Collembola | 8,186 | Chondrichthyes | 8,594 |
| Annelida | 1,551 | Branchiopoda | 5,016 | Unassigned class*** | 7,862 |
| Ascomycota | 330 | Ostracoda | 1,332 | Ascidiacea | 1,494 |
| Phaeophyceae | 246 | Diplopoda | 1,008 | Cladistia | 110 |
| Cnidaria | 101 | Pycnogonida | 898 | Thaliacea | 24 |
| Nematoda | 69 | Chilopoda | 703 | Appendicularia | 1 |
| Chaetognatha | 55 | Unassigned class** | 51 |  |  |
| Platyhelminthes | 53 | Merostomata | 41 |  |  |
| Bacillariophyta | 30 | Diplura | 34 |  |  |
| Nemertea | 12 | Remipedia | 20 |  |  |
| Brachiopoda | 10 | Protura | 19 |  |  |
| Porifera | 9 | Cephalocarida | 4 |  |  |
| Bryozoa | 4 |  |  |  |  |
| Xenacoelomorpha | 2 |  |  |  |  |
| Total sequences | 912,253 |  | 685,651 |  | 215,530 |

*Rhodophyta, Alveolata, Oomycetes

**Prionodiaptomus

***Ceratodontiformes, Coelacanthiformes, Crocodylia, Lepidosireniformes, Myxiniformes, Petromyzontiformes, Sphenodontia, Squamata, Testuines, Branchiostomidae

Table S2: CO1 Eukaryote v2 training set summary.

| **Training set** | **Number of taxa (all ranks)** | **Number of sequences** |
| --- | --- | --- |
| Whole training set | 123,145 | 912,621 |
| Arthropoda | 92,525 | 685,805 |
| Chordata | 27,475 | 215,727 |
| Outgroup taxa | 3,143 | 11,089 |

Table S3: Singletons in the CO1 Eukaryote v1 and v2 training sets.

| **CO1 Eukaryote v1 training set** | | | |
| --- | --- | --- | --- |
| **Rank** | **No. unique taxa** | **No. singletons** | **% Unique taxa that are singletons** |
| Superkingdom | 1 | - | - |
| Kingdom | 4 | 0 | - |
| Phylum | 18 | 0 | - |
| Class | 62 | 4 | 6.5 |
| Order | 389 | 27 | 6.9 |
| Family | 2,972 | 319 | 10.7 |
| Genus | 26,553 | 6,120 | 23.0 |
| **CO1 Eukaryote v2 training set** | | | |
| **Rank** | **No. unique taxa** | **No. singletons** | **% Unique taxa that are singletons** |
| Superkingdom | 1 | - | - |
| Kingdom | 4 | - | - |
| Phylum | 19 | 1 | 5.3 |
| Class | 63 | 5 | 7.9 |
| Order | 390 | 28 | 7.2 |
| Family | 2977 | 319 | 10.7 |
| Genus | 26,593 | 6,130 | 23.1 |
| Species | 93,112 | 30,958 | 33.2 |

Table S4: Suggested minimum bootstrap support cutoff values to recover 99% correct assignments with the CO1 Eukaryote v2 training set. This analysis is based on leave-one-out testing where singletons were excluded from the analysis. These cutoffs are based on 66.8% of the non-singleton sequences in the original training set. ‘N/A’, not applicable, refers to the inability to observe 99% correct taxonomic assignments.

| **Rank** | **500bp+** | **400bp** | **200bp** | **100bp** | **50bp** |
| --- | --- | --- | --- | --- | --- |
|  | **Minimum bootstrap support cutoff (%)** | | | | |
| Superkingdom | 0 | 0 | 0 | 0 | 0 |
| Kingdom | 0 | 0 | 0 | 0 | 0 |
| Phylum | 0 | 0 | 0 | 0 | 0 |
| Class | 0 | 0 | 0 | 0 | 30 |
| Order | 0 | 0 | 0 | 10 | 70 |
| Family | 0 | 0 | 20 | 20 | 70 |
| Genus | 30 | 30 | 30 | 30 | 80 |
| Species | N/A | N/A | N/A | N/A | N/A |
|  | **Reduction of sequences classified after applying minimum bootstrap support cutoff (%)** | | | | |
| Superkingdom | 0.0 | 0.0 | 0.0 | 0.0 | 0.0 |
| Kingdom | 0.0 | 0.0 | 0.0 | 0.0 | 0.0 |
| Phylum | 0.0 | 0.0 | 0.0 | 0.0 | 0.0 |
| Class | 0.0 | 0.0 | 0.0 | 0.0 | 2.4 |
| Order | 0.0 | 0.0 | 0.0 | 0.4 | 37.5 |
| Family | 0.0 | 0.4 | 1.4 | 3.5 | 59.2 |
| Genus | 1.1 | 1.7 | 2.5 | 7.1 | 72.7 |
| Species | N/A | N/A | N/A | N/A | N/A |

Table S5: Proportion of incorrect taxonomic assignments for Eukaryota phyla using the RDP classifier with the CO1 Eukaryote v1 training set. Calculated from leave-one-out testing results where singletons were included. We used a bootstrap support cutoff value of 70% for full length (500 bp+) CO1 sequences at the genus rank where indicated.

| **Eukaryote Phyla** | **No. misclassified sequences** | **Total sequences** | **% Misclassified**  **(No cutoff)** | **% Misclassified**  **(Cutoff)** |
| --- | --- | --- | --- | --- |
| Annelida | 41 | 1,551 | 2.6 | 0.5 |
| Arthropoda | 28,895 | 685,651 | 4.2 | 0.9 |
| Ascomycota | 5 | 330 | 1.5 | 0.3 |
| Bacillariophyta | 2 | 30 | 6.7 | 0.0 |
| Brachiopoda | 0 | 10 | 0.0 | 0.0 |
| Bryozoa | 1 | 4 | 25.0 | 0.0 |
| Chaetognatha | 4 | 55 | 7.3 | 5.5 |
| Chordata | 5,159 | 215,530 | 2.4 | 1.2 |
| Cnidaria | 17 | 101 | 16.8 | 7.9 |
| Echinodermata | 97 | 2,504 | 3.9 | 2.1 |
| Mollusca | 114 | 3,017 | 3.8 | 0.4 |
| Nematoda | 16 | 69 | 23.2 | 7.2 |
| Nemertea | 1 | 12 | 8.3 | 0.0 |
| Phaeophyceae | 10 | 246 | 4.1 | 0.4 |
| Platyhelminthes | 5 | 53 | 9.4 | 0.0 |
| Porifera | 1 | 9 | 11.1 | 0.0 |
| undef_undef_Eukaryota* | 105 | 3,079 | 3.4 | 1.3 |
| Xenacoelomorpha | 0 | 2 | 0.0 | 0.0 |
| Across all phyla | 34,473 | 912,253 | 3.8 | 1.0 |

*undef_undef_Eukaryota: Bangiophyceae, Dinophyceae, Florideophyceae, Oligohymenophorea, and Oomycetes.

Table S6: Proportion of incorrect taxonomic assignments for all Arthropoda classes using the RDP classifier with the CO1 Eukaryote v1 training set. Calculated from leave-one-out testing results where singletons were included. We used a bootstrap support cutoff value of 70% for full length (500 bp+) CO1 sequences at the genus rank where indicated.

| **Arthropoda Classes** | **No. misclassified sequences** | **Total sequences** | **% Misclassified (No cutoff)** | **% Misclassified (Cutoff)** |
| --- | --- | --- | --- | --- |
| Arachnida | 1,506 | 52,328 | 2.9 | 0.5 |
| Branchiopoda | 94 | 5,016 | 1.9 | 0.1 |
| Cephalocarida | 0 | 4 | 0.0 | 0.0 |
| Chilopoda | 79 | 703 | 11.2 | 1.3 |
| Collembola | 333 | 8,186 | 4.1 | 1.2 |
| Diplopoda | 74 | 1,008 | 7.3 | 1.1 |
| Diplura | 2 | 34 | 5.9 | 0.0 |
| Insecta | 24,945 | 561,841 | 4.4 | 0.9 |
| Malacostraca | 1,409 | 39,622 | 3.6 | 1.0 |
| Maxillopoda | 365 | 14,548 | 2.5 | 0.6 |
| Merostomata | 0 | 41 | 0.0 | 0.0 |
| Ostracoda | 30 | 1,332 | 2.3 | 0.5 |
| Protura | 2 | 19 | 10.5 | 0.0 |
| Pycnogonida | 46 | 898 | 5.1 | 0.1 |
| Remipedia | 1 | 20 | 5.0 | 0.0 |
| undef_Arthropoda* | 9 | 51 | 17.6 | 5.9 |
| Across all classes | 28,895 | 685,651 | 4.2 | 0.9 |

*undef_Arthropoda: Pauropoda, Symphyla

Table S7: Proportion of incorrect taxonomic assignments for all Chordata classes using the RDP classifier with the CO1 Eukaryote v1 training set. Calculated from leave-one-out testing results where singletons were included. We used a bootstrap support cutoff value of 70% for full length (500 bp+) CO1 sequences at the genus rank where indicated.

| **Chordata Classes** | **No. misclassified sequences** | **Total sequences** | **% Misclassified (No cutoff)** | **% Misclassified (Cutoff)** |
| --- | --- | --- | --- | --- |
| Actinopteri | 3,115 | 91,744 | 3.4 | 2.1 |
| Amphibia | 415 | 10,154 | 4.1 | 0.8 |
| Appendicularia | 1 | 1 | 100.0 | 100.0 |
| Ascidiacea | 63 | 1,494 | 4.2 | 1.1 |
| Aves | 791 | 25,150 | 3.1 | 1.2 |
| Chondrichthyes | 93 | 8,594 | 1.1 | 0.8 |
| Cladistia | 0 | 110 | 0.0 | 0.0 |
| Mammalia | 408 | 70,397 | 0.6 | 0.3 |
| Thaliacea | 2 | 24 | 8.3 | 8.3 |
| undef_Chordata* | 271 | 7,862 | 3.4 | 1.2 |
| Across all classes | 5,159 | 215,530 | 2.4 | 1.2 |

*undef_Chordata: Ceratodontiformes, Coelacanthiformes, Crocodylia, Lepidosireniformes, Myxiniformes, Petromyzontiformes, Sphenodontia, Squamata, Testudines, Branchiostomidae

Table S8: Proportion of incorrect taxonomic assignments for all Insecta orders using the RDP classifier with the CO1 Eukaryote v1 training set. Calculated from leave-one-out testing results where singletons were included. We used a bootstrap support cutoff value of 70% for full length (500 bp+) CO1 sequences at the genus rank where indicated.

| **Insecta Orders** | **No. misclassified sequences** | **Total sequences** | **% Misclassified (No cutoff)** | **% Misclassified (Cutoff)** |
| --- | --- | --- | --- | --- |
| Archaeognatha | 5 | 578 | 0.9 | 0.1 |
| Blattodea | 52 | 574 | 9.1 | 1.4 |
| Coleoptera | 6,672 | 89,484 | 7.5 | 1.1 |
| Dermaptera | 6 | 37 | 16.2 | 0.0 |
| Diptera | 4,503 | 118,896 | 3.8 | 0.8 |
| Embioptera | 24 | 64 | 37.5 | 4.7 |
| Ephemeroptera | 185 | 6,722 | 2.8 | 0.3 |
| Grylloblattodea | 1 | 1 | 100.0 | 0.0 |
| Hemiptera | 0 | 50,064 | 0.0 | 1.7 |
| Hymenoptera | 1,768 | 42,154 | 4.2 | 1.2 |
| Isoptera | 64 | 1,055 | 6.1 | 1.8 |
| Lepidoptera | 7,540 | 211,318 | 3.6 | 0.8 |
| Mantodea | 62 | 401 | 15.5 | 2.5 |
| Mantophasmatodea | 0 | 30 | 0.0 | 0.0 |
| Mecoptera | 17 | 285 | 6.0 | 1.8 |
| Megaloptera | 17 | 469 | 3.6 | 1.7 |
| Neuroptera | 125 | 1,545 | 8.1 | 2.3 |
| Odonata | 244 | 3,553 | 6.9 | 1.2 |
| Orthoptera | 338 | 6,885 | 4.9 | 1.9 |
| Phasmatodea | 87 | 786 | 11.1 | 1.0 |
| Phthiraptera | 15 | 1,103 | 1.4 | 0.0 |
| Plecoptera_Insecta | 72 | 2,679 | 2.7 | 0.1 |
| Psocoptera | 25 | 1,267 | 2.0 | 0.1 |
| Raphidioptera | 6 | 44 | 13.6 | 2.3 |
| Siphonaptera | 52 | 346 | 15.0 | 2.3 |
| Strepsiptera | 15 | 149 | 10.1 | 1.3 |
| Thysanoptera | 105 | 4,065 | 2.6 | 0.7 |
| Trichoptera | 528 | 17,276 | 3.1 | 0.3 |
| undef_Insecta* | 2 | 9 | 22.2 | 11.1 |
| Zoraptera | 0 | 2 | 0.0 | 0.0 |
| Across all orders | 22,530 | 561,841 | 4.0 | 0.9 |

*undef_Insecta: Lepidotrichidae, Lepismatidae, Nicoletiidae

Table S9: Proportion of incorrect taxonomic assignments for all Actinopteri orders using the RDP classifier with the CO1 Eukaryote v1 training set. Calculated from leave-one-out testing results where singletons were included. We used a bootstrap support cutoff value of 70% for full length (500 bp+) CO1 sequences at the genus rank where indicated.

| **Actinopteri Orders** | **No. misclassified sequences** | **Total sequences** | **% Misclassified (No cutoff)** | **% Misclassified (Cutoff)** |
| --- | --- | --- | --- | --- |
| Acanthuriformes | 11 | 827 | 1.3 | 0.6 |
| Acipenseriformes | 12 | 348 | 3.4 | 2.9 |
| Albuliformes | 3 | 70 | 4.3 | 1.4 |
| Alepocephaliformes | 15 | 161 | 9.3 | 3.7 |
| Amiiformes | 0 | 6 | 0.0 | 0.0 |
| Anabantiformes | 12 | 1,325 | 0.9 | 0.0 |
| Anguilliformes | 58 | 1,341 | 4.3 | 2.6 |
| Argentiniformes | 16 | 155 | 10.3 | 7.1 |
| Ateleopodiformes | 1 | 12 | 8.3 | 0.0 |
| Atheriniformes | 25 | 789 | 3.2 | 1.6 |
| Aulopiformes | 20 | 534 | 3.7 | 1.5 |
| Batrachoidiformes | 5 | 68 | 7.4 | 0.0 |
| Beloniformes | 21 | 517 | 4.1 | 1.7 |
| Beryciformes | 16 | 364 | 4.4 | 3.3 |
| Blenniiformes | 87 | 1,357 | 6.4 | 1.1 |
| Carangiformes | 44 | 2,041 | 2.2 | 1.6 |
| Centrarchiformes | 23 | 1,376 | 1.7 | 1.4 |
| Chaetodontiformes | 36 | 975 | 3.7 | 2.6 |
| Characiformes | 201 | 3,544 | 5.7 | 3.7 |
| Cichliformes | 292 | 2,007 | 14.5 | 11.5 |
| Clupeiformes | 70 | 1,767 | 4.0 | 2.9 |
| Cypriniformes | 593 | 13,285 | 4.5 | 3.4 |
| Cyprinodontiformes | 60 | 1,810 | 3.3 | 0.4 |
| Elopiformes | 0 | 90 | 0.0 | 0.0 |
| Ephippiformes | 0 | 98 | 0.0 | 0.0 |
| Esociformes | 1 | 348 | 0.3 | 0.0 |
| Gadiformes | 37 | 1,883 | 2.0 | 1.3 |
| Galaxiiformes | 3 | 258 | 1.2 | 0.0 |
| Gobiiformes | 169 | 4,371 | 3.9 | 2.0 |
| Gonorynchiformes | 0 | 58 | 0.0 | 0.0 |
| Gymnotiformes | 10 | 481 | 2.1 | 0.8 |
| Hiodontiformes | 0 | 24 | 0.0 | 0.0 |
| Holocentriformes | 8 | 376 | 2.1 | 1.9 |
| Istiophoriformes | 3 | 473 | 0.6 | 0.6 |
| Kurtiformes | 69 | 1,070 | 6.4 | 3.6 |
| Labriformes | 38 | 2,403 | 1.6 | 0.7 |
| Lampriformes | 7 | 53 | 13.2 | 7.5 |
| Lepidogalaxiiformes | 0 | 2 | 0.0 | 0.0 |
| Lobotiformes | 0 | 75 | 0.0 | 0.0 |
| Lophiiformes | 34 | 411 | 8.3 | 3.2 |
| Mugiliformes | 58 | 1,125 | 5.2 | 2.9 |
| Myctophiformes | 46 | 802 | 5.7 | 3.1 |
| Notacanthiformes | 3 | 119 | 2.5 | 0.8 |
| Ophidiiformes | 17 | 180 | 9.4 | 5.0 |
| Osmeriformes | 4 | 458 | 0.9 | 0.4 |
| Osteoglossiformes | 20 | 505 | 4.0 | 2.8 |
| Pempheriformes | 8 | 349 | 2.3 | 1.4 |
| Perciformes | 208 | 12,065 | 1.7 | 0.9 |
| Percopsiformes | 2 | 28 | 7.1 | 7.1 |
| Pholidichthyiformes | 1 | 1 | 100.0 | 0.0 |
| Pleuronectiformes | 78 | 2,475 | 3.2 | 1.6 |
| Polymixiiformes | 0 | 17 | 0.0 | 0.0 |
| Salmoniformes | 3 | 2,437 | 0.1 | 0.1 |
| Scombriformes | 56 | 3,290 | 1.7 | 1.3 |
| Semionotiformes | 0 | 68 | 0.0 | 0.0 |
| Siluriformes | 244 | 5,637 | 4.3 | 2.7 |
| Spariformes | 19 | 2,198 | 0.9 | 0.7 |
| Stomiiformes | 32 | 301 | 10.6 | 6.0 |
| Stylephoriformes | 0 | 5 | 0.0 | 0.0 |
| Synbranchiformes | 21 | 439 | 4.8 | 4.3 |
| Syngnathiformes | 65 | 1,587 | 4.1 | 1.4 |
| Tetraodontiformes | 37 | 1,531 | 2.4 | 1.8 |
| undef_Actinopteri* | 181 | 8,283 | 2.2 | 1.3 |
| Uranoscopiformes | 9 | 513 | 1.8 | 0.8 |
| Zeiformes | 3 | 178 | 1.7 | 0.6 |
| Across all orders | 3,115 | 91,744 | 3.4 | 2.1 |

*undef_Actinopteri: Ambassidae, Bathyclupeidae, Callanthiidae, Caproidae, Centropomidae, Cepolidae, Champsodontidae, Congrogadidae, Dichistiidae, Dinopercidae, Embiotocidae, Emmelichtyidae, Gerreidae, Grammatidae, Haemulidae, Hapalogenyidae, Lactariidae, Leptobramidae, Lutjanidae, Malacanthidae, Menidae, Monodactylidae, Moronidae, Opistognathidae, Parascorpididae, Plesiopidae, Polynemidae, Pomacanthidae, Pomacentridae, Priacanthidae, Pseudochromidae, Scatophagidae, Sciaenidae, Siganidae, Sillaginidae, Sphyraenidae, Symphysanodontidae, Toxotidae, Trichonotidae

Table S10: Number of class Insecta CO1 reference sequences in the 2013 GenBank-Genus Insecta training set^1^ and the current 2016 CO1 Eukaryote v1 training set.

| **Insecta Orders** | **2013** | **2016** |
| --- | --- | --- |
| Archaeognatha | 87 | 578 |
| Blattodea | 173 | 574 |
| Coleoptera | 27,325 | 89,484 |
| Dermaptera | 31 | 37 |
| Diptera | 21,838 | 118,896 |
| Embioptera | 30 | 64 |
| Ephemeroptera | 3,294 | 6,722 |
| Grylloblattodea | 1 | 1 |
| Hemiptera | 10,600 | 50,064 |
| Hymenoptera | 15,084 | 42,154 |
| Isoptera | 403 | 1,055 |
| Lepidoptera | 95,360 | 211,318 |
| Mantodea | 74 | 401 |
| Mantophasmatodea | 30 | 30 |
| Mecoptera | 34 | 285 |
| Megaloptera | 360 | 469 |
| Neuroptera | 434 | 1,545 |
| Odonata | 1,305 | 3,553 |
| Orthoptera | 3,292 | 6,885 |
| Phasmatodea | 681 | 786 |
| Phthiraptera | 179 | 1,103 |
| Plecoptera_Insecta | 1,016 | 2,679 |
| Psocoptera | 7 | 1,267 |
| Raphidioptera | 5 | 44 |
| Siphonaptera | 9 | 346 |
| Strepsiptera | 9 | 149 |
| Thysanoptera | 406 | 4,065 |
| Trichoptera | 8,257 | 17,276 |
| undef_Insecta* | 9 | 9 |
| Zoraptera | - | 2 |
| Across all orders | 190,333 | 561,841 |

*undef_Insecta: Lepidotrichidae, Lepismatidae, Nicoletiidae

Table S11: Top five Insecta orders with the most CO1 reference sequences 500 bp+ identified to the species rank in the GenBank nucleotide database.

| **2013** | **No. Seqs** | **2016** | **No. Seqs** |
| --- | --- | --- | --- |
| Lepidoptera | 95,360 | Lepidoptera | 211,318 |
| Coleoptera | 27,325 | Diptera | 118,896 |
| Diptera | 21,838 | Coleoptera | 89,484 |
| Hymenoptera | 15,084 | Hemiptera | 50,064 |
| Hemiptera | 10,600 | Hymenoptera | 42,154 |

Table S12: Bottom five Insecta orders with the least reference sequences 500 bp+ identified to the species rank in the GenBank nucleotide database.

| **2013** | **No. Seqs** | **2016** | **No. Seqs** |
| --- | --- | --- | --- |
| Grylloblattodea | 1 | Grylloblattodea | 1 |
| Raphidioptera | 5 | Zoraptera | 2 |
| Psocoptera | 7 | undef_Insecta | 9 |
| Strepsiptera | 9 | Mantophasmatodea | 30 |
| undef_Insecta* | 9 | Dermaptera | 37 |

*undef_Insecta: Lepidotrichidae, Lepismatidae, and Nicoletiidae

Table S13: Proportion of incorrect genus rank taxonomic assignments from the top 5 best-represented Insecta orders from two training sets has reduced from 2013^1^ to 2016. Results were summarized from leave-one-out testing of full length CO1 sequences (500 bp +), singletons included. No bootstrap support cutoff was used.

|  | **2013** | | **2016** | |
| --- | --- | --- | --- | --- |
| **Top Insecta Orders** | **No. sequences** | **% Incorrect** | **No. sequences** | **% Incorrect** |
| Hemiptera | 10,600 | 11.2 | 50,064 | 0 |
| Coleoptera | 27,325 | 9.1 | 89,484 | 7.5 |
| Diptera | 21, 838 | 7.9 | 118,896 | 3.8 |
| Hymenoptera | 15,084 | 6.3 | 42,154 | 4.2 |
| Lepidoptera | 95,360 | 3.9 | 211,318 | 3.6 |

Table S14: CO1 primers from the literature included in this study. Primers are listed in the order they would be found when reading from the 5’->3’ direction of the plus strand.

| **Primer Name** | **Orientation** | **Published Primer Sequence**  **(5'->3' orientation)*** | **References** |
| --- | --- | --- | --- |
| LCO1490 | Forward | GGTCAACAAATCATAAAGATATTGG | ^2^ |
| Lep-F1 | Forward | ATTCAACCAATCATAAAGATAT | ^3^ |
| Uni-MinibarF1 | Forward | TCCACTAATCACAARGATATTGGTAC | ^4^ |
| Fish_miniE_F | Forward | ACYAAICAYAAAGAYATIGGCAC | ^5^ |
| Uni-MinibarR1 | Reverse | GAAAATCATAATGAAGGCATGAGC | ^4^ |
| EPT-long-univR | Reverse | AARAAAATYATAAYAAAIGCGTGIAIIGT | ^6^ |
| A | Forward | GGIGGITTTGGIAATTGAYTIGTICC | ^7^ |
| ArF1 | Forward | GCICCWGAYATRGCITTYCCICG | ^8^ |
| ArF2 | Forward | GCICCIGAYATRGCITTYCCICG | ^8^ |
| ArF3 | Forward | GCICCRGAYATRGCITTYCCACG | ^8^ |
| ArF4 | Forward | GCICCCGATATRGCITTYCCYCG | ^8^ |
| ArF5 | Forward | GCICCIGAYATRKCITTYCCICG | ^8^ |
| ArF10 | Forward | CCWGATATAKCITWYCCICG | ^8^ |
| B | Forward | CCIGAYATRGCITTYCCICG | ^7^ |
| MF1 | Forward | GCTTTCCCACGAATAAATAATA | ^9^ |
| MLepF1-Rev | Reverse | CGTGGAAAWGCTATATCWGGTG | ^10^ |
| 230_R | Reverse | CTTATRTTRTTTATICGIGGRAAIGC | ^11^ |
| Fish_miniE_R | Reverse | CTTATRTTRTTTATICGIGGRAAIGC | ^5^ |
| mlCOIintF | Forward | GGWACWGGWTGAACWGTWTAYCCYCC | ^12^ |
| Fish_miniF_F | Forward | GGIACIGGITGRACIGTITAYCCYCC | ^5^ |
| C | Forward | GITGAACIGTITAYCCICC | ^7^ |
| MH-MR1 | Reverse | CCTGTTCCAGCTCCATTTTC | ^9^ |
| C_R | Reverse | GGIGGRTAIACIGTTCAICC | ^13^ |
| D | Reverse | CCTARIATIGAIGARAYICCIGC | ^7^ |
| ArR2** | Reverse | GTRATIGCICCIGCIARWACWGG | ^8^ |
| ArR3** | Reverse | GTRATWGCICCIGCTARWACWGG | ^8^ |
| ArR5** | Reverse | GTRATIGCICCIGCIARIACIGG | ^8^ |
| ArR6** | Reverse | GTRATIGCICCIGCYAAIACIGG | ^8^ |
| ArR7** | Reverse | GTRATTGCYCCIGCIARIACIGG | ^8^ |
| ArR9** | Reverse | GTRATIGCICCIGCWARIACWGG | ^8^ |
| E | Reverse | GTRATIGCICCIGCIARIAC | ^7^ |
| F | Reverse | CCIGCIGGRTCIAARAAIGAIGT | ^7^ |
| Fish_miniF_R | Reverse | CTTCAGGGTGICCGAARAATC | ^5^ |
| HCO2198 | Reverse | TAAACTTCAGGGTGACCAAAAAATCA | ^2^ |
| Lep-R1 | Reverse | TAAACTTCTGGATGTCCAAAAA | ^3^ |

*5’ -> 3’ orientation as forward primers would align to the plus strand and for reverse primers as they would align to the minus strand

**These reverse primer sequences were published in the 5’ -> 3’ orientation as they would align to the plus strand. Presented here in the 5’ -> 3’ orientation as they would align to the minus strand for consistency.

**References**

1. Porter, T. M. *et al.* Rapid and accurate taxonomic classification of insect (class Insecta) cytochrome c oxidase subunit 1 (COI) DNA barcode sequences using a naïve Bayesian classifier. *Mol. Ecol. Resour.* **14,** 929–942 (2014).

2. Folmer, O., Black, M., Hoeh, W., Lutz, R. & Vrijenhoek, R. DNA primers for amplification of mitochondrial cytochrome c oxidase subunit I from diverse metazoan invertebrates. *Mol. Mar. Biol. Biotechnol.* **3,** 294–299 (1994).

3. Hebert, P. D. N., Penton, E. H., Burns, J. M., Janzen, D. H. & Hallwachs, W. Ten species in one: DNA barcoding reveals cryptic species in the neotropical skipper butterfly Astraptes fulgerator. *Proc. Natl. Acad. Sci. U. S. A.* **101,** 14812–14817 (2004).

4. Meusnier, I. *et al.* A universal DNA mini-barcode for biodiversity analysis. *BMC Genomics* **9,** 214 (2008).

5. Shokralla, S., Hellberg, R. S., Handy, S. M., King, I. & Hajibabaei, M. A DNA Mini-Barcoding System for Authentication of Processed Fish Products. *Sci. Rep.* **5,** 15894 (2015).

6. Hajibabaei, M., Shokralla, S., Zhou, X., Singer, G. A. C. & Baird, D. J. Environmental Barcoding: A Next-Generation Sequencing Approach for Biomonitoring Applications Using River Benthos. *PLOS ONE* **6,** e17497 (2011).

7. Hajibabaei, M., Spall, J. L., Shokralla, S. & van Konynenburg, S. Assessing biodiversity of a freshwater benthic macroinvertebrate community through non-destructive environmental barcoding of DNA from preservative ethanol. *BMC Ecol.* **12,** 28 (2012).

8. Gibson, J. *et al.* Simultaneous assessment of the macrobiome and microbiome in a bulk sample of tropical arthropods through DNA metasystematics. *Proc. Natl. Acad. Sci.* **111,** 8007–8012 (2014).

9. Hajibabaei, M. *et al.* A minimalist barcode can identify a specimen whose DNA is degraded: BARCODING. *Mol. Ecol. Notes* **6,** 959–964 (2006).

10. Brandon-Mong, G.-J. *et al.* DNA metabarcoding of insects and allies: an evaluation of primers and pipelines. *Bull. Entomol. Res.* **105,** 717–727 (2015).

11. Gibson, J. F. *et al.* Large-Scale Biomonitoring of Remote and Threatened Ecosystems via High-Throughput Sequencing. *PLOS ONE* **10,** e0138432 (2015).

12. Leray, M. *et al.* A new versatile primer set targeting a short fragment of the mitochondrial COI region for metabarcoding metazoan diversity: application for characterizing coral reef fish gut contents. *Front. Zool.* **10,** 34 (2013).

13. Shokralla, S. *et al.* Massively parallel multiplex DNA sequencing for specimen identification using an Illumina MiSeq platform. *Sci. Rep.* **5,** 9687 (2015).
